# In Situ Driven Formation of Anatase/Brookite/Rutile Heterojunction N/TiO_2_ Nanocrystals as Sustainable Visible‐Light Catalysts

**DOI:** 10.1002/gch2.202400174

**Published:** 2024-09-19

**Authors:** Elias Assayehegn, Ananthakumar Solaiappan, Abraha Tadese Gidey, Gebremedhin Gebremariam Gebreegziabher, Tesfamariam Teklu Gebretsadik, Yonas Chebude, Esayas Alemayehu

**Affiliations:** ^1^ Materials Science and Technology Division National Institute for Interdisciplinary Science and Technology (NIIST‐CSIR) Thiruvananthapuram 695019 India; ^2^ National Centre for Catalysis Research and Department of Chemistry Indian Institute of Technology‐Madras Chennai 600036 India; ^3^ Department of Chemistry Mekelle University P.O. Box 231 Mekelle Ethiopia; ^4^ Faculty of Science Pavol Jozef Šafárik University Park Angelinum 9 Košice 04001 Slovakia; ^5^ Department of Chemistry Addis Ababa University P.O.Box 1176 Addis Ababa Ethiopia; ^6^ Faculty of Civil and Environmental Engineering Jimma University P.O.Box 378 Jimma Ethiopia

**Keywords:** doping, nanocrystals, phase transformation, photocatalyst, TiO_2_

## Abstract

Visible‐light active anatase/brookite/rutile (A/B/R) ternary N‐doped titania (N/TiO_2_) crystals are successfully prepared by a facile sol‐gel method using titanium butoxide and benign N‐dopant source, guanidinium chloride. Systematically varying the aging time (1, 4, 8, and 12 d), its influence on physicochemical properties of as‐obtained spherical heterojunction nanomaterials is studied. Detailed characterizations confirm that a substantial amount of anatase (88% to 50%) is transformed to rutile (2% to 38%) via intermediate brookite phase (9% to 25%) as the function of aging time; not only the A/B/R phase content of the samples is tuned by sol‐gel aging time of the precursors solution but also their optical‐response and methylene blue photocatalytic properties are profoundly dictated. Notably under visible‐light irradiation, the photostable rutile rich mesoporous A/B/R triphasic N/TiO_2_ (50% A, 12% B, 38% R) aged for 12 d demonstrates higher degradation activity (97%) with a faster degradation rate (0.033 min^−1^) than both lesser aged N/TiO_2_ and undoped titania. This enhancement is attributed to the synergistic effect of interstitial‐N‐doping and optimal A/B/R interfacial charge transfer that leads to higher light absorption, lower bandgap energy and well‐separated charge carriers. The current work provides a new perspective for designing highly active visible‐light heterostructure nanomaterials with controllable phase composition.

## Graphical abstract

In this study, tunable anatase/brookite/ rutile ternary N/TiO_2_ nanocrystals with well‐separated charge carriers are prepared by varying the aging time, often overlooked, which significantly influence the structure, morphology, optical response, and photoactivity. More importantly, this study reveals the importance of sol‐gel aging along with its in situ phase‐heterojunction formation, in addressing the inherent defects of TiO_2_ for energy and environmental remediation.

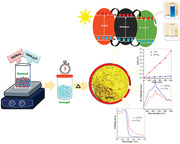

## 1 Introduction

As a global concern, the ever‐increasing energy crisis and environmental pollution have threatened humanity in the past several decades. In this regard, advanced oxidation processes have been extensively explored to remove persistence pollutants.^[^ ^1^ ^]^ Owning chemical inertness, photostability, and competitive price, titania or titanium dioxide (TiO_2_) is considered as the most promising photocatalyst in purifying contaminated water, soil and air.^[^ ^2^, ^3^ ^]^ This crystalline semiconductor predominately exists in anatase (A), rutile (R), and brookite (B) polymorphous. However, the particular material has faced critical foremost drawbacks for its practical applications, namely: 1) it has large bandgap energy, 3.2 eV, and only activates under UV light (that covers < 5% of the total sunlight); 2) it exhibits fast charge carrier recombination rate and poor solar conversion efficiency^[^ ^4^ ^]^ which lead to a low quantum yield, for instance 0.7% during 5 h light irradiation.^[^ ^3^ ^]^ In advancing its sunlight absorption, doping with hetero‐atom of metal and/or non‐metal has been proposed.^[^ ^3^ ^]^ Particularly, doping with anions (such as B, C, and S)^[^ ^5^ ^]^ has proven in alleviating the major aforementioned shortcomings unlike cations (like Pt, Pd, Ag, and Cd)^[^ ^6^ ^]^ which usually associate with photocorrosion and health problems.^[^ ^3^, ^4^ ^]^ Meanwhile, because of its ability to modify physicochemical properties, incorporating N into TiO_2_ matrix has received specific attention and several visible‐light active N‐doped TiO_2_ nanomaterials have been reported since the remarkable discovery of Asahi et al. on bandgap engineering of TiO_2_.^[^ ^7^, ^8^ ^]^

When N/TiO₂ is exposed to sunlight, its electrons can be excited, creating electron‐hole pairs (e^−^/h^+^) which are responsible for organic pollutant demineralization. This surface phenomenon should be done before electrons return; otherwise, e^−^/h^+^ will be recombined, one of the other major challenges in photocatalysis. Despite such considerable advancements, their capability of effectively separating the photogenerated charge carriers of e^−^/h^+^ (PCC) which boosts the quantum efficiency requires substantial improvement.^[^ ^7^, ^9^ ^]^ In recent years rather than preparing mono‐phase nanomaterials (of either anatase, rutile or brookite), preparing their mixed‐phase TiO_2_ nanostructures has been widely considered as the most promising strategy for quantum efficiency enhancement.^[^ ^10^, ^11^ ^]^ Consequently, these phase‐heterojunction materials have demonstrated a superior photocatalytic performance.^[^ ^4^ ^]^ In this context, the ideal example is Degussa P25 (a universal reference photocatalyst) which is made of 80% A and 20% R. Such A/R nanocomposites are extensively investigated,^[^ ^12^ ^]^ whereas few A/B^[^ ^10^ ^]^ and R/B^[^ ^13^ ^]^ dual phase N/TiO_2_ have been reported with notable photocatalytic and photo‐electrochemical performances.

Nevertheless, reports on A/B/R triphasic N/TiO_2_ nanocomposites are extremely rare due to the fact that their preparation techniques are challenging‐ follows multi‐step reaction under high temperature (usually > 600 °C) which damages their structure and texture properties. Wang et al.^[^ ^14^ ^]^ synthesized visible active N‐doped TiO_2_ material with 67% A, 26% B and 7% R using DMF as the N source, and solvothermal as post treatment; however these ternary nanoparticles were severed cause of poor crystallinity. Similarly, Preethi et al.^[^ ^15^ ^]^ synthesized A/B/R triphase N‐doped TiO_2_ nanotubes as sunlight active photocatalyst for water splitting with high quantum efficiency; however, a technically intense method of potentiostatic rapid breakdown anodization (Ti and Pt foils as the working and counter electrodes, respectively) is deployed along with a relatively toxic N‐source (hydrazine hydrate). Furthermore, the formation of oxygen vacancies, low N‐dopant level and usage of toxic hydrazine/ammonium fluoride as dopant source are the other challenges.^[^ ^16^ ^]^ Thus, developing a facile preparation method for tunable triphase N/TiO_2_ at lower temperature simultaneously minimizing the impact on the environment remains quite pivotal.

In an open literature survey, most investigations focused on the effect of N‐dopant concentration,^[^ ^15^ ^]^ temperature,^[^ ^17^ ^]^ surfactant type^[^ ^14^ ^]^ and solvent type; the obtained nanomaterials were aged mostly for 1–2 d.^[^ ^15^, ^17^ ^]^ Particularly, Ruzimuradov et al.^[^ ^18^ ^]^ prepared A/R crystalline N‐doped titania monoliths by ageing for 4 d of sequential solvent exchange and template‐based sol‐gel process followed by annealing at 700 °C under ammonia gas. They reported that A/R transformation took place between 600–700 °C, leading to low surface area, as low as 6 m^2^ g^−1^ with 50% visible‐light degradation performance. They did not disclose the role of ageing on physicochemical properties of N/TiO_2_. The effect of aging time on crystal structure, phase composition, morphology, optical response and photocatalytic activity of N/TiO_2_ nanomaterials is overlooked.

Guanidinium chloride (GUA) is a benign amine molecule with high protein denaturing character. In spite of its huge N‐content, it is barely used as N‐dopant source; limited works have been reported so far.^[^ ^19^ ^]^ In the present work with a special focus on aging synthesis approach, we report for the first time its usage in preparing various A/B/R heterojunction N/TiO_2_ nanoparticles. Thus, ultimate objective of this study was to examine the effect of ageing on physicochemical properties and preparation of ternary N/TiO_2_ using GUA The as‐synthesised materials are denoted as N‐*y* where *y* stands for number of aging days, and a control sample as N‐0 without the addition of N‐dopant. A deep characterization of the obtained catalysts was performed via XRD, XPS, EPR, HRTEM, FESEM, DRS, PL, BET, EDS, Raman, FTIR, and TGA. Their photodegradation activity was assessed against MB solution under visible light. More importantly, the decisive attributing factors for the recorded improved degradation performance along with a possible photocatalytic mechanism were thoroughly discussed.

## 2 Results and Discussion

Guanidinium chloride, CH_6_N_3_Cl, was selected as N‐dopant source due to huge N (44% by weight) released during its decomposition (300–380 °C). The undoped and N‐doped TiO_2_ powders were successfully prepared by hydrolysis/condensation of Ti(OBu)_4_ and GUA, and crystallized at 400 °C in air. In order to probe the effect of aging time on crystal structure, texture, optical response and photocatalytic properties, the resultant samples were subjected to different sol‐gel age time (**Table** **1**).

**Table 1:** Summaries of synthesis parameters, powder XRD data, specific surface area, and pore volume of A/B/R N/TiO_2_ nanomaterials.

Catalysts	Ti(OBu)_4_ [mmol]	GUA [mmol]	Aging [d]	Phase composition [%]			^a)^ *E*_g_ [eV]	^b)^ *S*_BET_ [m^2^ g^−1^]	^c)^ *D*_BJH_ [nm]
				Anatase	Rutile	Brookite			
N‐0	11.38	0	12	88	3	9	3.16	84	9.6
N‐1		11.38	1	74	2	24	3.21	78	10.2
N‐4		11.38	4	68	8	24	3.19	83	4.9
N‐8		11.38	8	67	8	25	3.13	82	5.9
N‐12		11.38	12	50	38	12	2.92	79	5

^a)^ *E*_g_: bandgap energy;

^b)^ *S*_BET_: specific surface area;

^c)^ *D*_BJH_: pore size.

### 2.1 XRD and Raman Analysis

**Figure 1:**
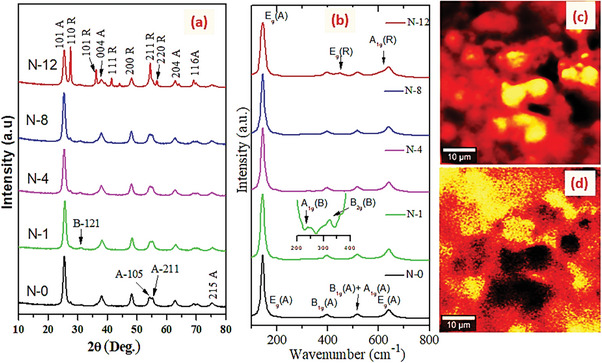
a) Powder XRD patterns and b) Raman spectra of as‐obtained N/TiO_2_; the anatase c) and rutile d) confocal Raman images of N‐12 at anatase and rutile peak positions of 145 cm^−1^ and 445 cm^−1^, respectively (A=Anatase, B=Brookite, R=Rutile).

**Figure** **1**a depicts the XRD patterns of the various TiO_2_ based nanomaterials. Their sharp diffraction peaks at 2*θ* of 25.4°, 37.8°, 48.3°, 54.2°, and 62.9° respond to (101), (004), (200) (105) and (204) crystal planes of the anatase phase (JCPDS: 21‐1272), respectively. Whereas the peaks at 27.5° (110), 36.3° (101), 41.3° (111), 44.2° (210), and 54.3° (211) are assigned to the rutile phase (JCPDS: 21‐1276). Meanwhile the presence of a third phase, brookite was confirmed by the peak at 2*θ* of 30.8° (121), the only typical peak that not overlapped with any of A and/or R peaks. However, the XRD Rietveld refinements (Figure S1, Supporting Information) revealed the remaining brookite peaks. It can be noted that the XRD data not only confirmed that all the materials are composed of A/B/R triphase, but also their phase composition is varied with respective aging time (Table 1).

**Scheme 1:**
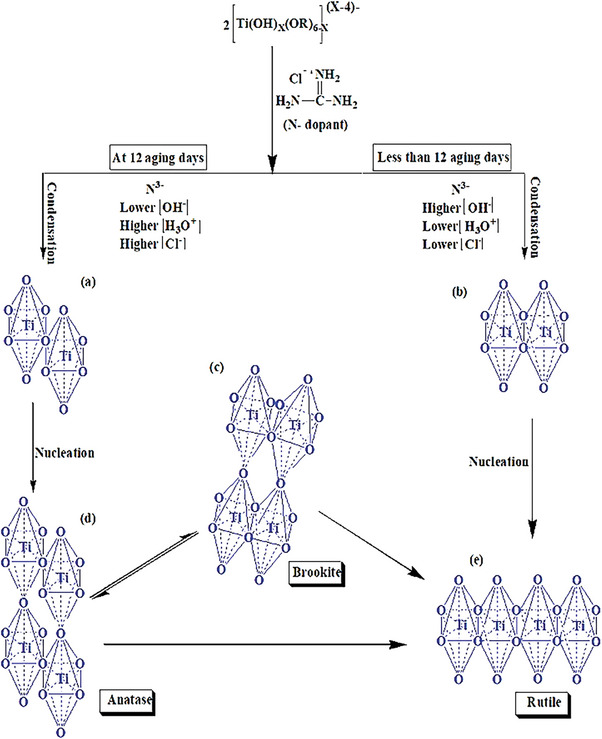
Proposed phase transformation mechanism for A/B/R ternary of as‐obtained materials.

It is clearly seen that as the aging time increases, the amount of anatase and brookite is declined while rutile significantly increased‐ indicating either anatase to rutile (A→R), a major one, or brookite to rutile, a minor, phase transformation has taken place; detail explanations are followed. Ti‐precursor readily hydrolyses to Ti‐hydroxyl‐alkoxide (Ti‐OH‐OR) intermediates which eventually seed TiO_6_ octahedron units. As per the assembly manner of these TiO_6_ building blocks, various TiO_2_ crystals are nucleated and grew during hydrolysis/condensation steps. For instance, A and R are formed when the octahedrons are shared by their corners and edges, respectively (**Scheme** **1**a,b) whereas in brookite, both corners and edges are shared (Scheme 1c). Nevertheless, the initial organization mode of TiO_6_ depends on the condensation/nucleation of Ti‐OH‐OR intermediate which is considerably influenced by cumulative effect of synthesis conditions (such as type of preparation method, type/amount of precursors, pH, temperature and gas type), dopant nature (its type, concentration, charger and ionic radius), intermediate phase formation and particle size.^[^ ^20^, ^21^ ^]^ In the present study, by systematically comparing and contrasting the pristine and N‐doped materials, and N‐doped materials among themselves, it is reasonably possible to point out that the phase transformation is occurred because of three significant motives: the presence of N^3−^ dopant, involvement of in situ H_3_O^+^/Cl^−^ ions, and variation of aging time. For instance, N‐0 and N‐12 were passed through the same synthesis conditions (aged for 12 d) except the latter was with GUA, the N‐dopant source. However their respective A and R content significantly differ; A covers 88% in the undoped N‐0 sample, yet it is reduced to 50% in doped N‐12. Whereas R is increased from 3% of N‐0 to 38% of N‐12 with minimal brookite increment (9% to 12%) (Table 1). This outcome strongly suggests that the A→R transformation is occurred due to the introduction of N^3−^ dopant which has larger ionic radius and more negative charge than the crystal O^2‐^ anion that would initiate bond rupturing, ionic rearrangement and structure reorganization for the transformation by disrupting the lattice orientation and charge density of TiO_2_ matrix.

Meanwhile, comparing the N‐doped TiO_2_ photocatalysts among themselves, the variation of aging time has brought a profound effect on their colloids physical properties during hydrolysis, polymerization and coarsening processes. For instance, it is observed that on 12^th^ day of aging, the pH was lowered to 0.5 (due to the in situ dissociated H_3_O^+^ and Cl^−^ species of the N‐source) which initially was 1.2 (Table S1, Supporting Information). In such a highly acidic medium, the concentration of coordinating OH^−^ for the Ti‐OH‐OR intermediate formation is low. This consecutively favors the edge‐shared binding, facilitating the nucleation of rutile and brookite. Moreover, the in situ dissociated Cl^−^ of N/TiO_2_ samples (which found positive against AgNO_3_ test) might incorporate in the Intermediate structure and leads to a formation new and more symmetric Ti‐chloro‐complex by which rutile/brookite are again preferred. Thus as the result of GUA dissociation not only changes the medium to more acidic but also its in situ Cl^−^ ions are incorporated in intermediate structure; similar findings have been reported.^[^ ^22^ ^]^ It can be considered that the more the aging time the sample has, the more time it would have for H_3_O^+^/Cl^−^ disassociation and bond rapturing‐forming process. Consecutively, anatase and brookite are reduced (74% of N‐1 to 50% of N‐12) and (24% to 12%), respectively while rutile is significantly increased (2% to 38%). Thus, this increment of R by the expense of A and B decrement strongly suggests that both anatase and brookite are transferred to rutile up on aging (Scheme 1c–e). Moreover, having shared edges and corners, brookite is structurally intermediate between A and R. Thus the interconversion of A→R via brookite becomes much easier (involves simple displacement of atoms into adjacent sites) than directly; such deduction is in accordance with literatures.^[^ ^23^, ^24^ ^]^

Furthermore, we investigated the effect of calcination on the A→R transformation beside the aging time. By comparing the XRD patterns of before and after calcination of N‐12 sample (Figure S2, Supporting Information), it is observed that the annealing has affected the crystal structure of N/TiO_2_; uncalcined N‐12 nanoparticles were anatase monophase with less degree of crystallinity whereas after its calcination they became A/B/R triphase with higher crystallinity nature. Thus, not only aging time but also the annealing has influenced the transformation. It is important to note that the 400 °C A→R transformation temperature in N/TiO_2_ nanocrystals is significantly lower than a previous report.^[^ ^18^ ^]^ Meanwhile, regardless of the respective aging time, the Debye‐Scherrer crystallite size of anatase, brookite and rutile of N/TiO_2_ nanomaterials are generally similar with average of 9.4, 23, and 54 nm, respectively (Table S2, Supporting Information).

In other structure analysis, the Raman spectra (Figure 1b) show that all the materials have peaks of tetragonal anatase modes at 144 (E_1g_), 197 (E_2g_), 399 (B_1g_), 514 (A_1g_ + B_1g_) and 639 (E_3g_) cm^−1^. Moreover, they have additional two rutile modes at 445 (E_1g_) and 612 (A_1g_) cm^−1^ with typical Raman modes of brookite at 245 (A_1g_) and 320 (B_1g_) cm^−1^.^[^ ^25^ ^]^ Thus the Raman spectra further proved the coexistence of the ternary phase in as‐prepared N‐doped materials, which is in accordance with XRD results. Notably, the confocal Raman images of N‐12 at specific peak positions of 144 cm^−1^ and 445 cm^−1^ (Figure 1c,d) indicate that its surface is covered dominantly by A and R phases.

### 2.2 SEM, TEM, and EDS analysis

**Figure 2:**
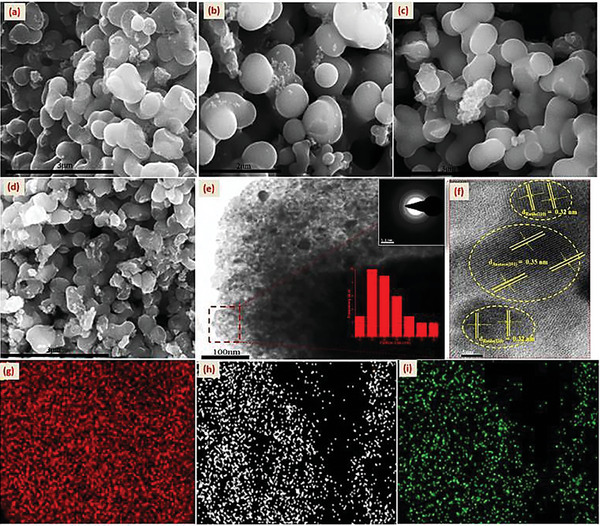
FESEM of as‐obtained nanomaterials a) N‐1, b) N‐4, c) N‐8, d) N‐12; HRTEM of N‐12 e,f) with inset SAED and particle size; its elemental mapping of g) Ti, h) O, i) N.

The field emission‐SEM images (**Figure** **2**a–d) show that as‐obtained titania‐based catalysts exhibit similar morphology, consisting of aggregated spherical nanoparticles (200–500 nm) which assembled and grew into coral like structures due to rapid hydrolysis/condensation of Ti(OBu)_4_. However, the TEM image of the N‐12 (Figure 2e) reveals that those aggregates of N/TiO_2_ nanoparticles comprise primary particles in a diameter range of 15–20 nm (Figure 2e inset), which is in agreement with XRD data. Its SAED image (Figure 2e inset) shows concentric ring patterns which specify its high crystallinity nature. More importantly, the HRTEM image (Figure 2f) clearly indicates the presence of lattice fringe of neighbor nanoparticles of 0.32 and 0.35 nm which well‐match with (110) and (101) planes of rutile and anatase, respectively. Thus, the HRTEM further confirms the coexistence of well‐defined mixed phase heterojunction; though, due to the low amount presence, the brookite phase is not clearly seen in the image. Meanwhile, the EDS result (Figure S3, Supporting Information) reveals that the titania powders are effectively doped with N and its percentage (4‐7 at%) is increased as the function of aging days. Moreover, the elemental mapping (Figure 2g–i) proves that the doped‐N is homogeneously distributed in the crystal structure. In addition, the FTIR spectra of as‐obtained (Section S1 and Figure S4, Supporting Information) further confirm the incorporation of N. Thus, guanidinium chloride is an effective N‐dopant source and by optimizing the aging time, the incorporation of N species in the TiO_2_ crystal can be tuned.

### 2.3 N_2_ Sorption Studies

**Figure 3:**
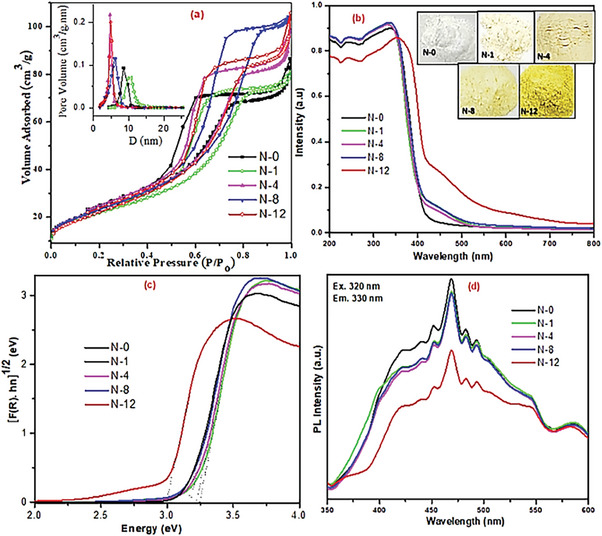
a) N_2_ sorption isotherms with inset BJH pore size distribution. b) DRS spectra with inset photographs of doped powders. c) Tauc plot; d) PL spectra of as‐obtained TiO_2_ based catalysts.

**Figure** **3**a depicts the N_2_ adsorption–desorption isotherms and pore size distribution of as‐obtained N/TiO_2_ materials and is summarized in Table 1. It can be seen that all nanomaterials, viz., N‐0, N‐1, N‐4, N‐8, and N‐12, showed similar type‐IV sorption isotherms, indicating their mesoporous nature with monomodal pore size distribution. At low values of relative pressures, *P*/*P*_o_, the adsorption volume is increased, attributing to monolayer and multilayer adsorption in micro/mesopores. The onset of hysteresis was observed at a relative pressure of 0.47 beyond which a sudden adsorption volume increment was observed, representing a capillary condensation inside the uniform size pores. Meanwhile, the N/TiO_2_ nanomaterials have lower specific surface area (*S*_BET_) than unmodified powder by 2–6 m^2^ g^−1^ as the aging day increases (Table 1), due to the grain growth and agglomeration as discussed in the SEM section. Such effect of *S*_BET_ reduction as the result of N introduction was also reported.^[^ ^26^ ^]^ However, since rutile‐TiO_2_ nanostructures are formed at high temperature, they usually associate with low surface area, but these as‐prepared rutile rich A/B/R N/TiO_2_ display larger *S*_BET_ with narrow pore size distribution than reported data.^[^ ^27^, ^28^, ^29^ ^]^ It can fairly presume that these catalysts demonstrate high photodegradation due to their higher exposed active surface area for surface adsorption/reaction of pollutants. It can be noted (Figure 3a inset) that the pore volume increased while pore size decreased as the function of aging time.

### 2.4 DRS Analysis

The DRS spectra of pure TiO_2_ and A/B/R N‐doped TiO_2_ (Figure 3b with inset photographs of doped‐powders) display a very broad absorption band at 415 nm (UV region) for unmodified sample, and a roughly shoulder‐tail absorption bands <440 and 440–600 nm for N/TiO_2_ materials, corresponding to electron excitation from valence band (VB) and newly formed energy level (due to N‐doping) to the conduction band (CB) of TiO_2_, respectively. Unlike the UV‐active undoped titania, all the yellowish doped catalysts, viz., N‐1, N‐4, N‐8, and N‐12, showed an extended tail absorption towards visible region, boosting their effective solar light utilization. It is noted that the more the aging time, the more light absorption at a higher wavelength; N‐12 displays the highest visible absorption. Similarly, from their Tauc plot (Figure 3c), all N/TiO_2_ have lower bandgap energy (*E*_g_) than the undoped sample; N‐12 specifically records the lowest, 2.92 eV (Table 1). Thus, as a result of effective N‐doping, such bathochromic (red shift) absorption and lower *E*_g_ are observed and consequently enhance the optical response of N/TiO_2_ photocatalysts.

### 2.5 PL Analysis

Having high surface area and light absorption, and low bandgap energy are not the only determinants for photocatalytic activity, but also the nature of photoexcited e^−^/h^+^. For any surface reaction, once the e^−^/h^+^ are exited, they should not recombine. In this context, PL emission measurement is the key technique to understand how the fate of PCC phenomenon has been affected by the introduction of N. Figure 3d represents the PL spectra of TiO_2_ nanomaterials aged at various days at *λ*_exc._ = 320 nm excitation; as it can be clearly observed, all samples exhibit a broad emission signal center at 475 nm (2.68 eV) that is ascribed to PCC recombination.^[^ ^30^ ^]^ A similarly study done by Moreira et al.^[^ ^31^ ^]^ on doped‐TiO_2_ nanostructures, those intermediate levels were attributed to shallow defects due to oxygen vacancies. The undoped TiO_2_ showed a broadband with greater PL intensity (the black line in Figure 3d), inferring the material presents many defects responsible for increasing the PCC recombination. Conversely, all the N/TiO_2_ powders demonstrate lower PL peak intensity than the pure TiO_2_ (Figure 3d). This strongly deduces that the introduction of the N species in the crystal of TiO_2_ did not create trapping sites that shorten lifespan of e^−^/h^+^, in contrary lowers the charge recombination rate and extends their lifetime. Besides this doping benefits, having A/B/R heterojunction plays a significant role in separating the e^−^/h^+^ in the N/TiO_2_ materials due to the their migration in opposite directions across the ternary phase, though the direction is controversial.^[^ ^32^ ^]^ Similar results were previously reported for mixed phase TiO_2_ materials.^[^ ^31^, ^33^, ^34^ ^]^ Comparing the PL results amongst N/TiO_2_ samples, it is noted that the recombination rate is decreased as aging time increased. Unlike the other A/B/R N/TiO_2_, the rutile rich N‐12 exhibits the most PL quenching (27% lower than N‐0). This is one of the main reasons why the particular nanomaterial displayed the highest MB degradation performance under visible light irradiation, which will be discussed later.

### 2.6 XPS and EPR Analysis

**Figure 4:**
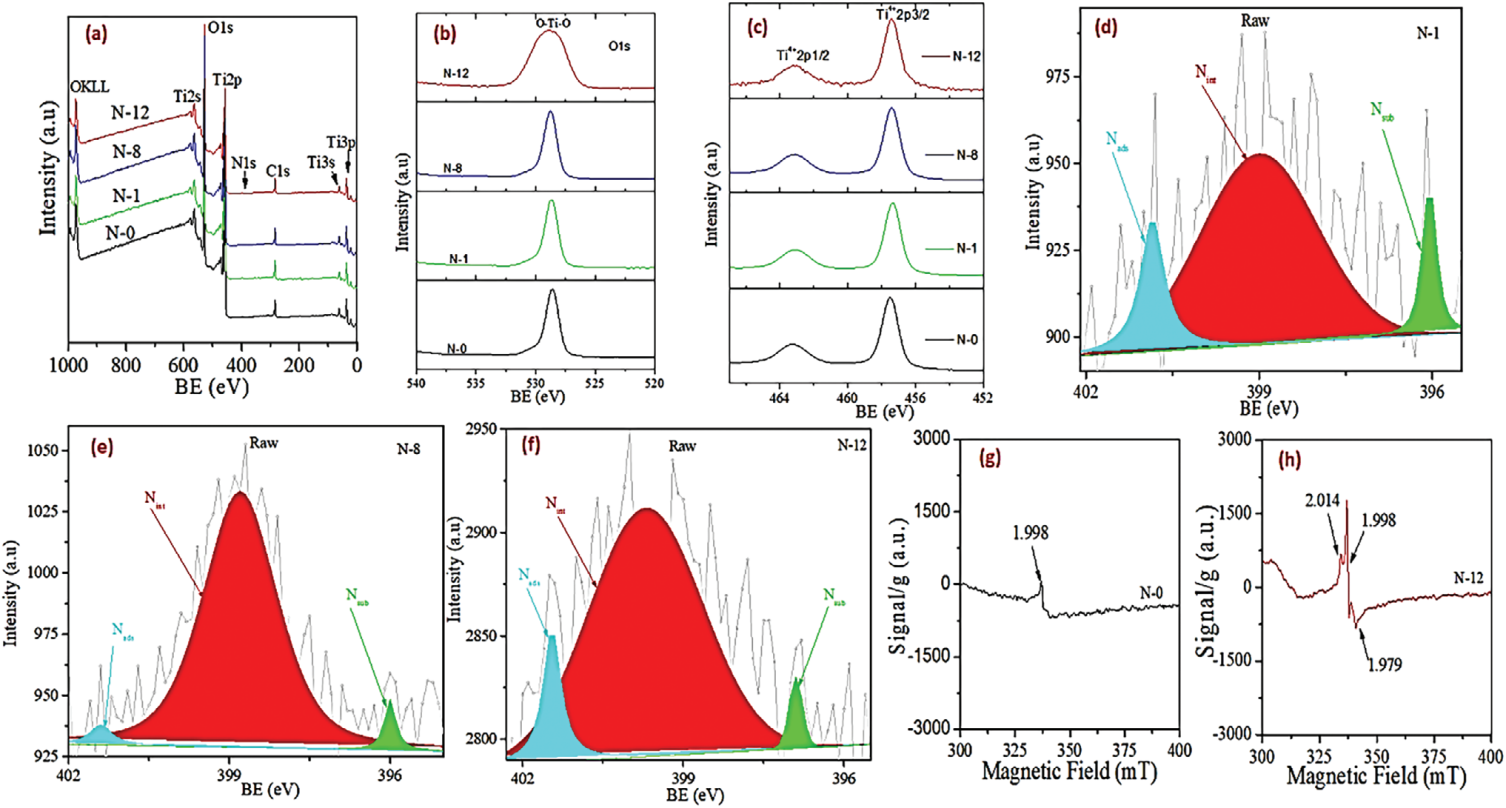
a) XPS survey, b) Ti 2p spectra and c) O 1s spectra of as‐obtained nanomaterials; N 1s spectra of d) N‐1, e) N‐8 and f) N‐12; EPR spectra of g) N‐0, h) N‐12.

In investigation the surface chemical composition and species type/state which affect the surface reaction, photocatalysis, XPS is a powerful tool; it was deployed for the as‐synthesized samples. A fully scanned XPS spectrum (**Figure** **4**a) confirmed the existence of Ti, O, C (due to remnant organic precursors) and N elements. More importantly, the successful incorporation of N in the TiO_2_ matrix has been revealed once again. From the high‐resolution O 1s spectra (Figure 4b), the peak at a binding energy (BE) of 529.1 eV corresponds to crystal lattice oxygen. However N‐12 extends a broader BE near 531.2 eV, inferring the presence of oxygen vacancies and/or surface hydroxides.^[^ ^35^ ^]^ According to Ti 2p spectra of as‐obtained materials (Figure 4c), the doublet peak at 457.5 and 463.3 eV are indexed to Ti^4+^ 2p_3/2_ and Ti^4+^ 2p_1/2_, respectively.^[^ ^36^ ^]^ However, comparing with the undoped sample, the BE for Ti 2p of N‐12 is relatively shifted to lower BE which indicates electron density increment on Ti, cause of its binding to N anions which partially transfer electron to Ti, comparing to electron‐withdrawing O. This is an indication that N is effectively doped in titania, which consistent with earlier report.^[^ ^30^ ^]^

On the other hand, it is recognized that the doped‐N can be located either at substitutional and/or interstitial sites, however assigning and identifying their respective N 1s BE and individual contribution for visible driven photocatalytic activity in N/TiO_2_ nanostructure are still controversial.^[^ ^4^, ^7^ ^]^ In many cases, the BE in the range of 395.5–397 eV is assigned to substitutional N (N_sub_), which sited in oxygen lattice position, while 398–400 eV to interstitial N (N_int_). In this study, the high‐resolution of N 1s peaks of N‐1, N‐8 and N‐12 (Figure 4d–f) are clearly seen in the range 398–402 eV with peak area ratio of 3:4:8, respectively. Moreover, each of these broad peaks is deconvoluted into three parts for better understanding the type of N species. Their dominant N 1s peak centered at 399.6 eV is attributed to N_int_, however this value is higher than of TiN (that has a typical Ti‐N‐Ti linkage), appears at 397.5 eV. This might infer that the doped‐N in the TiO_2_ is attached with a more electronegative O atom which consequently withdraws electron density from N atom. Thus, the attachment of N_int_ can be regarded as Ti‐N‐O and/or O‐Ti‐N. Evidently, this is supported by lower Ti 2p BE (as discussed earlier) and previous reports.^[^ ^30^ ^]^ Additionally, these materials have smaller second and third deconvoluted peaks at 396.2 and 401.5 eV which correspond to the N_sub_ and surface adsorbed NO_x_ species (N_ads_), respectively.^[^ ^37^ ^]^ Thus according to N 1s spectra, N/TiO_2_ nanomaterials have three N species: *N*_int_, *N*_sub,_ and *N*_ads_; it is imperative, at this point, to deal further on their individual role towards photodegradation activity, the other debatable matter of such nanostructures. Details for the deconvoluted N 1s peak area and percentage coverage of each N species of the samples are displayed (Table S3, Supporting Information); however the calculated percentage of *N*_int_, *N*_sub,_ and *N*_ads_ of each catalysts viz., N‐1, N‐8 and N‐12, is presented (**Table** **2**).

**Table 2:** XPS N content and various N species percentage of as‐obtained catalysts.

Catalysts	^a)^ *N* [at%]	^b)^ *N*_int_ [%]	^b)^ *N*_sub_ [%]	^b)^ *N*_ads_ [%]
N‐1	0.58	80.87	7.38	11.75
N‐8	0.69	93.82	3.44	2.74
N‐12	0.98	90.65	2.65	6.70

^a)^ *N* % analyzed by XPS;

^b)^ Percentage of individual fitted peak area to the total fitted N1s peak area

It can be noted that all the doped materials exhibit a significant amount of *N*_int_ (>80%) over *N*_sub_ (<8%); correlating N species with photodegradation performance, N‐12 with 90.6% *N*_int_ and 2.7% *N*_sub_ demonstrates a 97% degradation performance. Similarly, N‐8 and N‐1 with 93.8% and 80.9% *N*_int_ content display 69% and 71% activity, respectively. Thus, this result strongly suggests that interstitial N dominantly initiates the photodegradation, which is in accordance with previous studies, though the performance depends on various factors. such as crystal nature/phase composition, light absorption, surface area, particle size and bandgap energy.^[^ ^38^, ^39^, ^40^ ^]^

Meanwhile, the paramagnetic species identification for visible light absorption and photodegradation improvement in as‐prepared materials was performed. According to the EPR spectrum of the unmodified N‐0 sample (Figure 4g), low oxygen radical density is indicated as the result of the weak signal at g value of 1.998, whereas N‐12 shows additional two strong EPR lines at g values of 2.014 and 1.978 (Figure 4h), ascribing to a high concentration of surface O_2_^−^ and Ti^3+^, respectively. Nevertheless, no paramagnetic‐N species is detected, confirming the above discussed *N*_int_ is a diamagnetic entity.

### 2.7 Evaluation of Photocatalytic Activity

**Figure 5:**
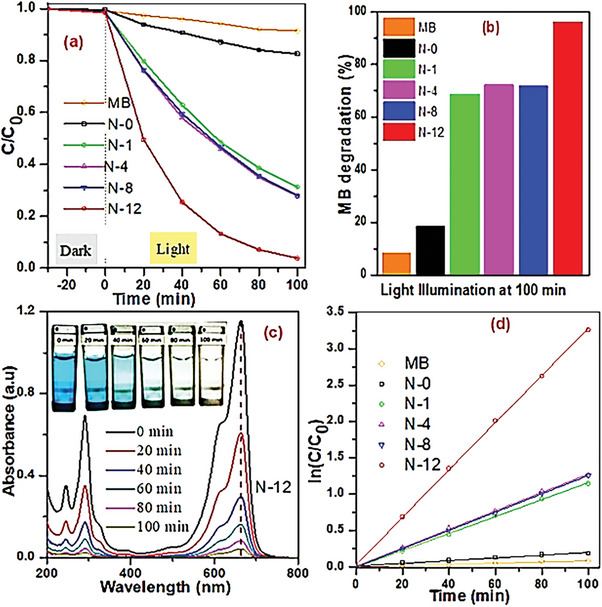
a) MB degradation rate; b) MB photodegradation percentage of as‐obtained catalysts under 100 min visible light; c) UV–Vis spectra of MB solutions over N‐12 with inset photographs before and after the final degradation; d) pseudo first‐order kinetics of as‐obtained samples.

The photodegradation of as‐prepared catalysts (50 mg) was evaluated against a model pollutant MB (10 mg L^−1^) under fluorescent lamp (112 W, 3.2 mW cm^−2^) and its result is illustrated (**Figure** **5**). It is clearly observed (Figure 5a) that the degradation of all as‐obtained catalysts is increased as the function of irradiation time, specifically the doped materials, viz., N‐1, N‐4, N‐8 and N‐12, have showed higher performance than the undoped titania. Notably N‐12 demonstrates the highest performance (97%) within 100 min of visible‐light illumination (Figure 5b); it is important to note that the degradation percentage is increased with aging time, from 67% of N‐1 to 97% of N‐12. Nevertheless, N‐0 and blank MB have degraded 18% and 8%, due to concomitant oxygen deficiency and photosensitivity, respectively. Evidently up on the illumination over N‐12 (Figure 5c), not only the characteristic peak of MB is quickly diminished but also its blue color photobleached (Figure 5c and inset photos). According to their pseudo first order MB photodegradation kinetics (Figure 5d and Table S4, Supporting Information), it can be noted that N‐12 has recorded the highest rate constant of 0.0325 min^−1^ which is 17.4 times faster than the undoped material, even higher than similar N/TiO_2_ photocatalysts reported elsewhere (Table S5, Supporting Information).

This enhanced photocatalytic activity is ascribed to its red shift light absorption, lower bandgap energy and recombination rate, and optimal A/B/R mixed phase. Furthermore, the particular material displays a significant recyclability, in five consecutive recycles (Figure S5, Supporting Information) and thermal stability (Section S2 and Figure S6, Supporting Information) without affecting its structure and morphology (Figure S7, Supporting Information). Moreover, from terephthalic acid fluorescence (TPA‐PL) experiment (Figure S8, Supporting Information), to determine the amount of generated reactive radicals, the particular photocatalyst, A/B/R N‐12, produces a huge amount of free hydroxyl radicals within the visible‐light irradiation period. It is known these highly reactive radicals are the primary oxidizing species for majority of photocatalytic activity; thus, the degradation performance becomes higher with prolonging irradiation time. Overall, the MB degradation of the as‐prepared powders is significantly dictated by sol‐gel aging time; as the aging time increases, not only enhances the anatase to rutile phase transformation and visible‐light absorption but also well separated PCC are generated.

### 2.8 Photocatalytic Mechanism

**Scheme 2:**
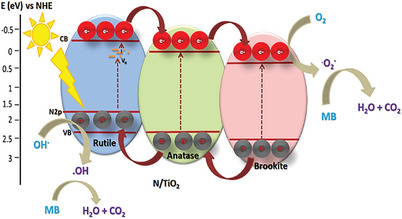
Proposed light induced photocatalytic mechanism over A/B/R N/TiO_2_ catalyst.

The MB photodegradation mechanism is well documented (Figure S9, Supporting Information); based on the above deployed analyses (XRD, DRS, XPS, EPR, PL and TPA‐PL), a possible photocatalytic mechanism over tri‐phase heterojunction N/TiO_2_ is illustrated (**Scheme** **2**). It is observed that the introduction of N, into TiO_2_ crystal structure, modifies the electronic alignment, forming new states (N 2p) on the top of the VB (O 2p), that in return narrows the bandgap and induces visible light absorption. Thus, up on visible‐light illumination, the N/TiO_2_ nanomaterials easily produce numerous PCC in which the photogenerated electrons are quickly transferred to the CB of TiO_2_ leaving the h^+^ in midgap. These e^−^/h^+^ will eventually disperse onto the surface of the mesoporous catalysts. Moreover, such N‐TiO_2_ creates of oxygen vacancy (O_v_) energy level, trapping the electrons with consequent improvement of the PCC efficiency.^[^ ^41^, ^42^ ^]^ More importantly, having A/B/R heterojunction in N/TiO_2_ separates these PCC in such a way that the e^−^ transfer from rutile to brookite via anatase whereas the h^+^ migrates in the opposite way.^[^ ^10^, ^16^ ^]^ Consequently, this prolongs the lifetime of charge carriers along with suitable opportunity to participate in MB degradation being exposed to surface. To understand more about the degradation mechanism, we further studied band alignment of the as‐obtained materials with respect to O_2_/O_2_^•−^ and ^•^OH/H_2_O potentials. The CB and VB positions of N/TiO_2_ were calculated (Section S3, Supporting Information) and found −0.145 and 2.77 eV for CB and VB of N‐12, respectively. Since the energy, of CB, is more negative to O_2_/O_2_^•−^ potential (0.13 eV), the accessible e^−^ would effectively reduce adsorbed O_2_ to highly oxidative superoxide radicals (O_2_^•−^) for MB decomposition. On the other hand, the photoexcited h^+^ could participate in the degradation process by directly oxidizing the water polluting MB molecules and/or reacting with water/hydroxyl to produce another powerful ^•^OH radicals (2.72 eV), which was detected by TPA‐PL probe. Thus O_2_^•−^, ^•^OH and h^+^ species are accountable for the MB mineralization under visible light irradiation.

## 3 Conclusion

Highly crystalline mesoporous A/B/R mixed phase N‐doped TiO_2_ nanomaterials were prepared using a benign N‐source, guanidinium chloride via sol‐gel method. Varying the aging days significantly influences the phase transformation/composition, surface chemistry, optical response and MB degradation performance of as‐prepared N/TiO_2_ powders. The rutile rich tri‐phase (50% A, 12% B, 38% R) photocatalyst aged for 12 d exhibits the highest (97%) and fastest (0.0325 min^−1^, 17 times faster than the bare TiO_2_) MB degradation within 100 min visible‐light irradiation along with appreciable recyclability, structure and thermal stability. This outstanding activity is attributed to the synergistic effect of N‐doping and its optimal A/B/R heterojunction which consequently led to higher light absorption capacity and specific surface area, lower bandgap energy and charge carriers recombination as well as higher interstitial doped‐N. Such aging synthesis approach of visible‐light active tri‐phase N/TiO_2_ crystals sets a benchmark in designing nanomaterials in different fields.

## 4 Experimental Section

### Synthesis of N‐Doped TiO_2_ Nanomaterials

The preparation of doped/undoped TiO_2_ nanocrystals was based on the pervious reported sol‐gel protocol;^[^ ^43^, ^44^ ^]^ titanium (IV) butoxide (Ti(OBu)_4_, 98%, Sigma Aldrich) and guanidinium chloride (GUA, 98%, Sigma Aldrich) were used as Ti and N‐dopant sources, respectively. Typically, 11.4 mmol Ti(OBu)_4_ was added stepwise to ethanoic solution (30 mL, ethanol/water with 5:1 ratio) while stirring vigorously, to obtain Ti‐solution. In separate beaker, an equimolar GUA was added to acidic‐ethanoic solution (10 mL ethanol and 0.3 mL conc. HNO_3_) to form N‐dopant solution. To the prepared Ti‐solution, the N‐dopant mixture was slowly added drop by drop and stirred vigorously for 2 h until a homogeneous suspension was obtained. The milky sol was sealed in vials and aged for certain (1–12) d at room temperature (Table 1). Then the resultant white gel was dried at 80 °C overnight after being washed and centrifuged with ethanol/water several times. To obtain the final yellowish powder, it was annealed at 400 °C for 4 h at 1 °C min^−1^ in air. The as‐synthesized materials were denoted as N‐*y* where *y* stands for number of aging days. Thereafter a control sample was prepared, designated as N‐0, following the same procedure without the addition of N‐dopant source. Milli‐Q water (18.5 MΩ cm^−1^) was used during the whole experiment.

### Characterization

Detailed characterization techniques/parameters are provided supplementing information (Section S4, Supporting Information). Briefly, the structure and phase purity of the as‐obtained materials were studied by powder X‐ray diffraction (XRD) using a X‐ray diffractometer (Bruker D8 Advance) and confocal Raman Microscopy (Witec‐Alpha 300R, Germany) at 633 nm laser excitation. Material analysis using diffraction (MAUD) software was used for structural XRD refinement. Besides, Fourier transform infrared spectroscopy (FTIR) is recorded on a Perkin Elmer Spectrum Two spectrometer using attenuated total reflectance mode. Morphology of as‐obtained catalysts was studied by high‐resolution scanning electron microscopy, FESEM (Quanta 400 FEG) and high‐resolution transmission electron microscopy, HRTEM, (Tecnai G^2^, The Netherlands). Their elemental composition and mapping were analyzed by energy dispersive X‐ray spectroscopy, EDS (Carl Zeiss, Germany) and X‐ray photoelectron spectroscopy, XPS (ULVAC‐PHI‐500, USA) using curve fitting software XPSSPEAK 41. Moreover, the electronic paramagnetic resonance (EPR) spectra were carried out by JES‐FA200 spectrometer. The diffuse reflectance spectra (DRS) and photoluminescence (PL) spectra of the ternary photocatalysts were recorded by UV‐Vis (JASCO V‐650) and fluorescence (JASCO FP‐6500) spectrophotometers. N_2_ adsorption‐desorption isotherms were collected (ASAP 2020 BET analyzer) using Brunauer‐Emmett‐Teller (BET) and Barrett‐Joyner‐Halenda (BJH) methods. Their thermal behaviours were analyzed by a thermal analyser (STA 7300, Hitachi).

### Photocatalytic Evaluation

The MB photodegradation experiments were conducted in a photoreactor equipped with 16 fluorescent lamps (112 W, 3.2 mW cm^−2^) under a continuous stirring. Typically, as‐obtained powder (50 mg) was dispersed over aqueous MB solution (100 mL, 10 mg L^−1^); prior each reaction, the suspension was ultrasonicated and stirred for 5 and 30 min, respectively in the dark at room temperature to ensure the adsorption‐desorption equilibrium establishment. Then, under visible‐light irradiation, 2.5 mL aliquots of the suspension were withdrawn every 20 min interval and the catalysts were separated by centrifugation. No oxygen gas was bubbled into the suspension. The reusability test was assessed; the as‐synthesized material was recovered via successive washing, centrifuging and drying. All recycle photoreactions were followed the same aforementioned experimental conditions. The change in MB concentration of the supernatant solution was measured by UV–vis spectrophotometer (UV‐2401 PC, Shimadzu, Japan) for calculating degradation efficiency; the photodegradation kinetics is described as pseudo first order by Langmuir‐Hinshelwood model. Meanwhile, to determine the amount of hydroxyl radicals (^•^OH) created by the catalyst, terephthalic acid (TPA, 98%, Sigma‐Aldrich) fluorescence experiment (TPA‐PL) was performed. By trapping of free ^•^OH, a non‐fluorescent TPA is changed to highly fluorescent 2‐hydroxyterephthalic acid (TPA‐OH), which exhibits a characteristic PL peak located at ≈425 nm. The above procedure was followed for TPA‐PL except the MB dye was replaced with 5 × 10^−4^ molar of TPA and 2 × 10^−3^ m of NaOH which enhances TPA solubility. The PL spectra of TPA‐OH were recorded using a spectrofluorometer (Cary Eclipse‐Varian, The Netherlands) at an excitation wavelength of 315 nm.

The authors have cited additional references within the Supporting Information.^[^ ^14^, ^26^, ^27^, ^45^, ^46^, ^47^, ^48^, ^49^, ^50^, ^51^, ^52^, ^53^, ^54^, ^55^, ^56^, ^57^, ^58^ ^]^

## Conflict of Interest

The authors declare no conflict of interest.

## Supporting information



Supporting Information

## Data Availability

The data that support the findings of this study are available in the supplementary material of this article.
