# Supplementary material for: In Situ Driven Formation of Anatase/Brookite/Rutile Heterojunction N/TiO2 Nanocrystals as Sustainable Visible‐Light Catalysts
Source: Glob Chall. 2024 Sep 19;8(10):2400174. doi: 10.1002/gch2.202400174 (PMC11469765; doi:10.1002/gch2.202400174)
Supplement: Supplementary file 1 — Supporting Information [file GCH2-8-2400174-s001.docx]

**Supplementary Material**

***In-situ* driven formation of anatase/brookite/rutile heterojunction N/TiO_2_ nanocrystals as sustainable visible-light catalysts**

Elias Assayehegn,^[1,2,3,4,]*^ Ananthakumar Solaiappan,^[1]^ Abraha Tadese Gidey,^[3]^ Gebremedhin Gebremariam,^[3]^ Tesfamariam Teklu Gebretsadik,^[3]^ Yonas Chebude,^[5]^ and Esayas Alemayehu^[6]*^

^[1]^ Materials Science and Technology Division, National Institute for Interdisciplinary Science and Technology (NIIST-CSIR), Thiruvananthapuram, India-695019

^[2]^ National Centre for Catalysis Research, and Department of Chemistry, Indian Institute of Technology-Madras, Chennai-600036, India

^[3]^ Department of Chemistry, Mekelle University, P.O. Box 231, Mekelle, Ethiopia

^[4]^ Faculty of Science, Pavol Jozef Šafárik University, Park Angelinum 9, 04001 Košice, Slovakia

^[5]^ Department of Chemistry, Addis Ababa University, P.O.Box 1176, Addis Ababa; Ethiopia

^[6]^ Faculty of Civil and Environmental Engineering, Jimma University, Jimma, P.O.Box 378; Ethiopia

^*^Corresponding Authors

E-mail: [assaye98@gmail.com](mailto:assaye98@gmail.com) E. Assayehegn, and [esayas16@yahoo.com](mailto:esayas16@yahoo.com) E. Alemayehu

Contents

**List of Figures**

[Figure S1. Retrieved refinement XRD patterns of as-prepared pure and N-doped TiO_2_ (a) N-12 with its magnified fitting curve, (b) N-0, (c) N-1, (d) N-4, (e) N-8: Anatase (A), Rutile (R), Brookite (B), and goodness of fitting (S). S4](file:///C:\Users\ASUS\Desktop\RSC,2019\RSC-MAN-Supporting%20Information@2021.docx#_Toc82248671)

[Figure S2. XRD patterns before and after annealing N-12 sample. S5](file:///C:\Users\ASUS\Desktop\RSC,2019\RSC-MAN-Supporting%20Information@2021.docx#_Toc82248672)

[Figure S3. EDS of (a) undoped TiO_2_, N-0, (b) N-1 (c) N-4, (d) N-8 and (e) N-12. S7](file:///C:\Users\ASUS\Desktop\RSC,2019\RSC-MAN-Supporting%20Information@2021.docx#_Toc82248673)

[Figure S4. FTIR spectra of as-obtained N-0, N-8 and N-12 nanomaterials. S8](file:///C:\Users\ASUS\Desktop\RSC,2019\RSC-MAN-Supporting%20Information@2021.docx#_Toc82248674)

[Figure S5. Recycle test of N-12 photocatalyst under 100 min visible-light illumination. S11](file:///C:\Users\ASUS\Desktop\RSC,2019\RSC-MAN-Supporting%20Information@2021.docx#_Toc82248675)

[Figure S6. TGA of as-obtained nanomaterials. S12](file:///C:\Users\ASUS\Desktop\RSC,2019\RSC-MAN-Supporting%20Information@2021.docx#_Toc82248677)

[Figure S7. FESEM image (a) and XRD pattern (b) of N-12 photocatalyst after three MB degradation tests; A=Anatase, R=Rutile, B=Brookite. S12](file:///C:\Users\ASUS\Desktop\RSC,2019\RSC-MAN-Supporting%20Information@2021.docx#_Toc82248676)

[Figure S8. Fluorescence intensity of 2-hydroxyterephthalic acid solution for N-12 sample under different visible-light irradiation S13](file:///C:\Users\ASUS\Desktop\RSC,2019\RSC-MAN-Supporting%20Information@2021.docx#_Toc82248678)

[Figure S9. Photodegradation mechanism of MB over surface of photocatalyst. S14](#_Toc82248679)

**List of Tables**

[Table S1. pH and Cl- ion test results of undoped/N-doped nanomaterials as per their aging time. S5](#_Toc82249886)

[Table S2. Crystal size and lattice parameters of as-obtained undoped/N-doped TiO_2_ materials. S6](#_Toc82249887)

[Table S3. Deconvoluted N 1s peak areas and their respective percentage of N-1, N-8 and N-12. S9](#_Toc82249888)

[Table S4. The reaction rate constant (k) of MB degradation in the presence of various pure and N/TiO_2_ samples under visible-light illumnation S9](#_Toc82249889)

[Table S5. Comparison of synthesis and photocatalytic performance of various N/TiO_2_ nanomaterials. S10](#_Toc82249890)

**List of Sections**

[S1. FTIR spectra of as-prepared nanomaterials S7](#_Toc169950507)

[S2. The thermogravimetric analysis of as-prepared TiO_2_ based catalysts S11](#_Toc169950508)

[S3. Calculating CB/VB positions of as-prepared N/TiO_2_ catalyst S14](#_Toc169950509)

[S4. Characterization Techniques S14](#_Toc169950510)

[References S15](#_Toc169950511)


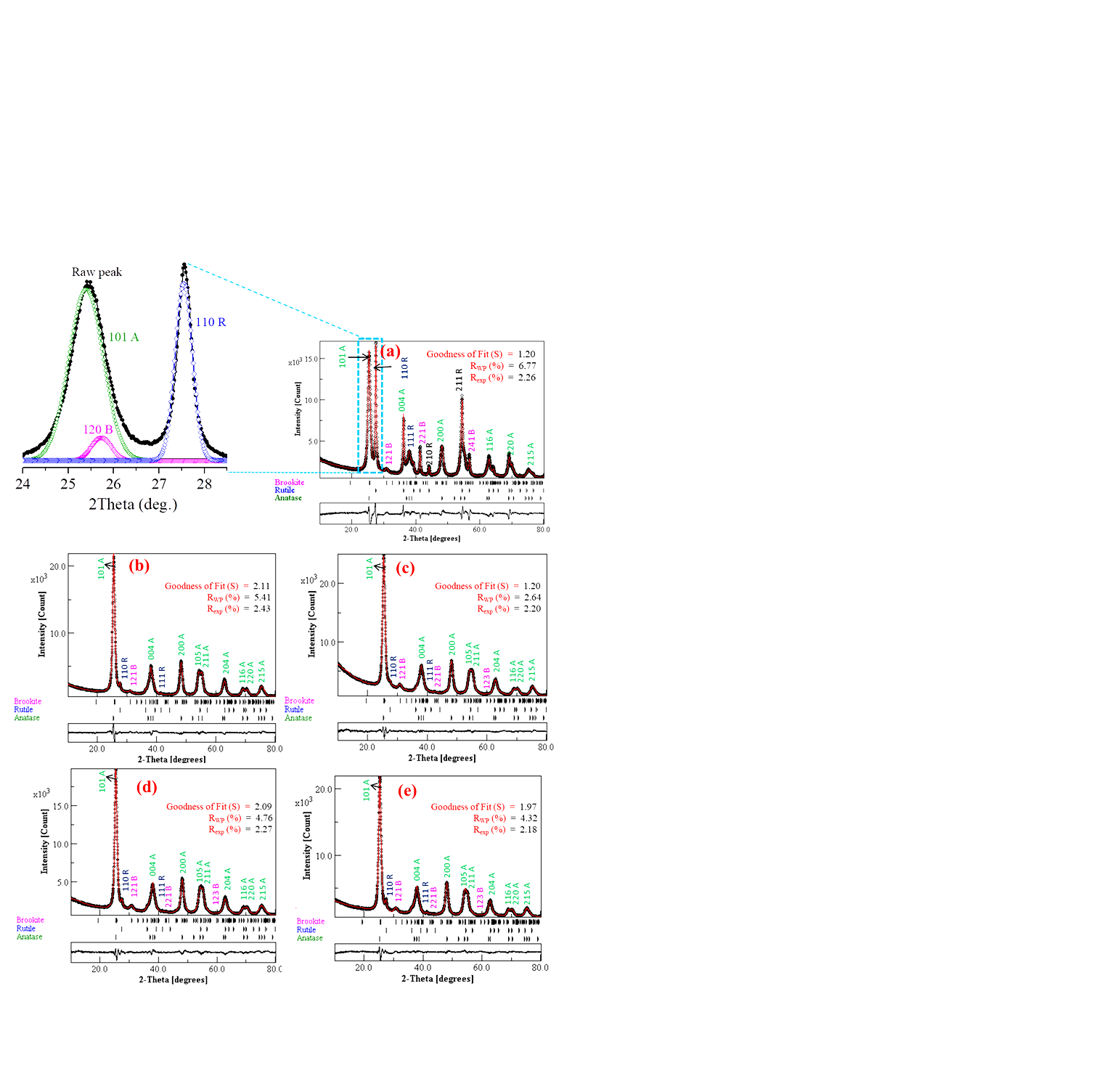


Figure S1. Retrieved refinement XRD patterns of as-prepared pure and N-doped TiO_2_ (a) N-12 with its magnified fitting curve, (b) N-0, (c) N-1, (d) N-4, (e) N-8: Anatase (A), Rutile (R), Brookite (B), and goodness of fitting (S).

Table S1. pH and Cl^-^ ion test results of undoped/N-doped nanomaterials as per their aging time.

| Catalysts | pH | | | | AgNO_3_ Test (for Cl^-^ ion) |
| --- | --- | --- | --- | --- | --- |
|  | 1^st^ day | 4^th^ day | 8^th^ day | 12^th^ day |  |
| N-0 | 5.6 | 5.5 | 5.4 | 5.5 | - |
| N-1 | 1.1 |  |  |  | + |
| N-4 | 1.2 | 1.03 |  |  | + |
| N-8 | 1.2 | 1.02 | 0.90 |  | + |
| N-12 | 1.1 | 1.02 | 0.87 | 0.5 | + |


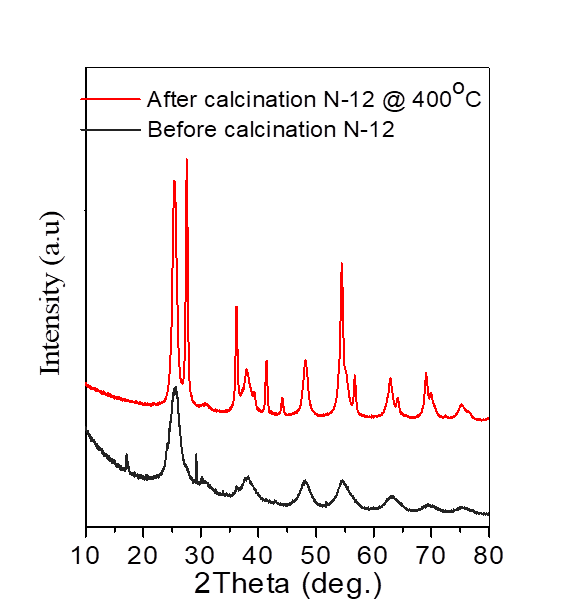


Figure S2. XRD patterns before and after annealing N-12 sample.

Table S2. Crystal size and lattice parameters of as-obtained undoped/N-doped TiO_2_ materials.

| Catalysts | Anatase | | Brookite | | Rutile | |
| --- | --- | --- | --- | --- | --- | --- |
|  | Crystal size  (nm) | Lattice parameters  (Å) | Crystal size  (nm) | Lattice parameters  (Å) | Crystal size  (nm) | Lattice parameters  (Å) |
| N-0 | 13 | a = 3.77617(13)  c = 9.4795(8) | 27 | a = 9.078(4)  b = 5.3843(17)  c = 5.151(3) | 56 | a = 4.5704(11)  c = 2.9528(17) |
| N-1 | 9 | a = 3.7779(2)  c = 9.4688(5) | 23 | a = 9.095(4)  b = 5.4268(13)  c = 5.1568(14) | - | a = 4.575(2)  c = 2.960(3) |
| N-4 | 9 | a = 3.7810(2)  c = 9.4614(9) | 23 | a = 9.195(9)  b = 5.427(3)  c = 5.165(2) | 52 | a = 4.5951(12)  c = 2.9523(19) |
| N-8 | 9 | a= 3.78192(17)  c= 9.4827(11) | 23 | a = 9.118(8)  b = 5.454(3)  c = 5.178(2) | 53 | a = 4.5882(6)  c = 2.9493(7) |
| N-12 | 10 | a = 3.7775(2)  c = 9.4924(11) | 22 | a = 9.006(13)  b = 5.499(5)  c = 5.131(5) | 59 | a = 4.5825(15)  c = 2.9534(16) |


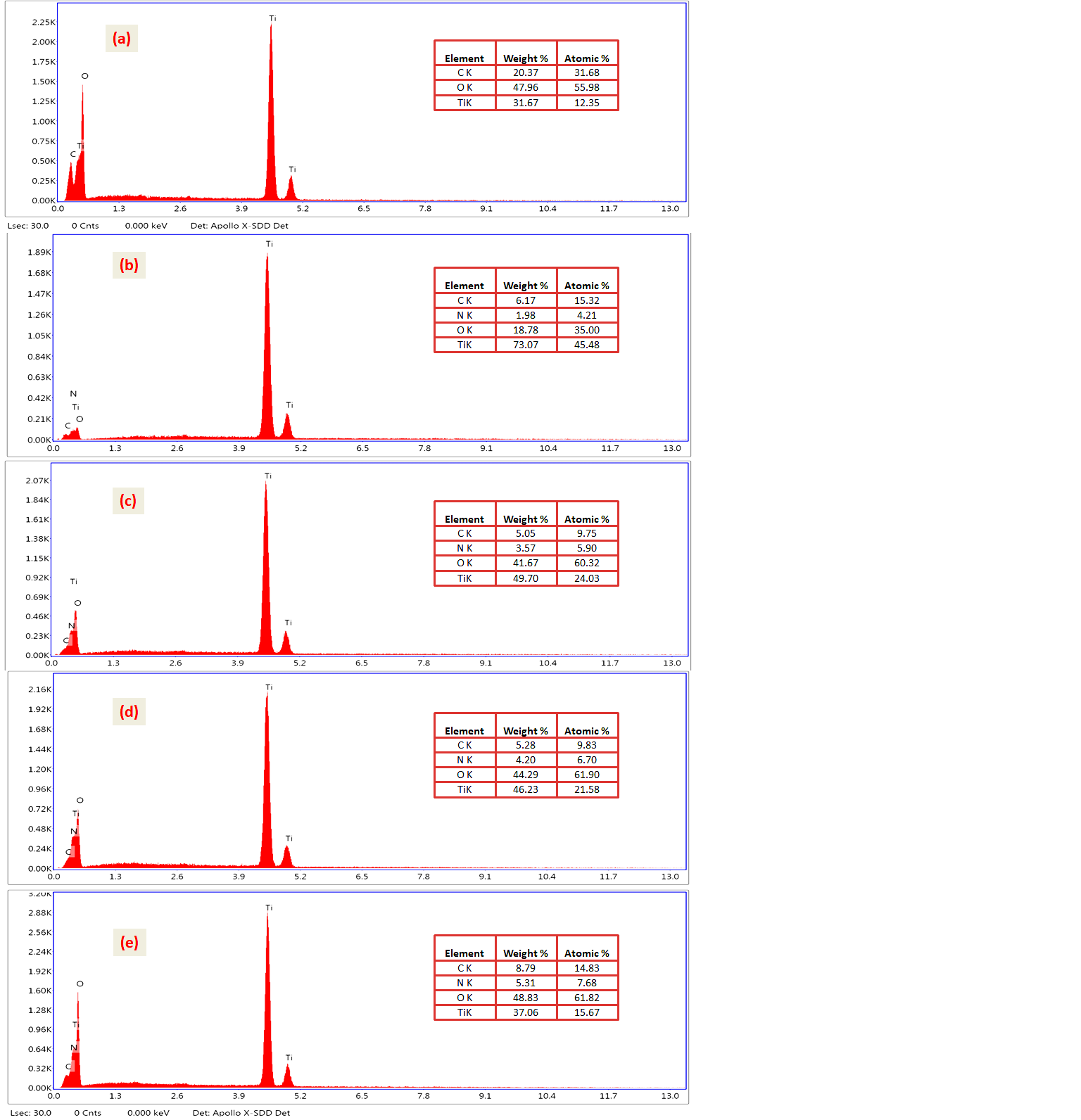


Figure S3. EDS of (a) undoped TiO_2_, N-0, (b) N-1 (c) N-4, (d) N-8 and (e) N-12.

# S1. FTIR spectra of as-prepared nanomaterials

The below FTIR spectra of pure and N/TiO_2_ (Figure S4) display the presence of incorporated functional group. The intensive and broad band at around 3240 cm^-1^ is due to presence of Ti-OH groups and physisorbed/chemisorbed water molecules along with their bending frequency appeared at 1630 cm^-1^.^[1,2]^ It has been also observed peaks corresponding to carbon impurities including saturated and unsaturated C-H bonds in the range of 2300-3300 cm^-1^, likely from precursors and solvents present on the sample surface.^[2]^ The peak at 740 cm^-1^ is attributed to Ti-O and Ti-O-Ti bridging stretching modes.^[3]^ Moreover comparing with the pure titania, the N/TiO_2_ showed new peaks at 3700 cm^-1^, 1740 cm^-1^ and 520 cm^-1^ are ascribed for the presence of N-H hydrogen bonding possibly due to H_2_O/NH_3_, N-H bending vibrations and Ti-N bond, respectively.^[4]^ This strongly suggests that N species have been incorporated into the TiO_2_ lattice.


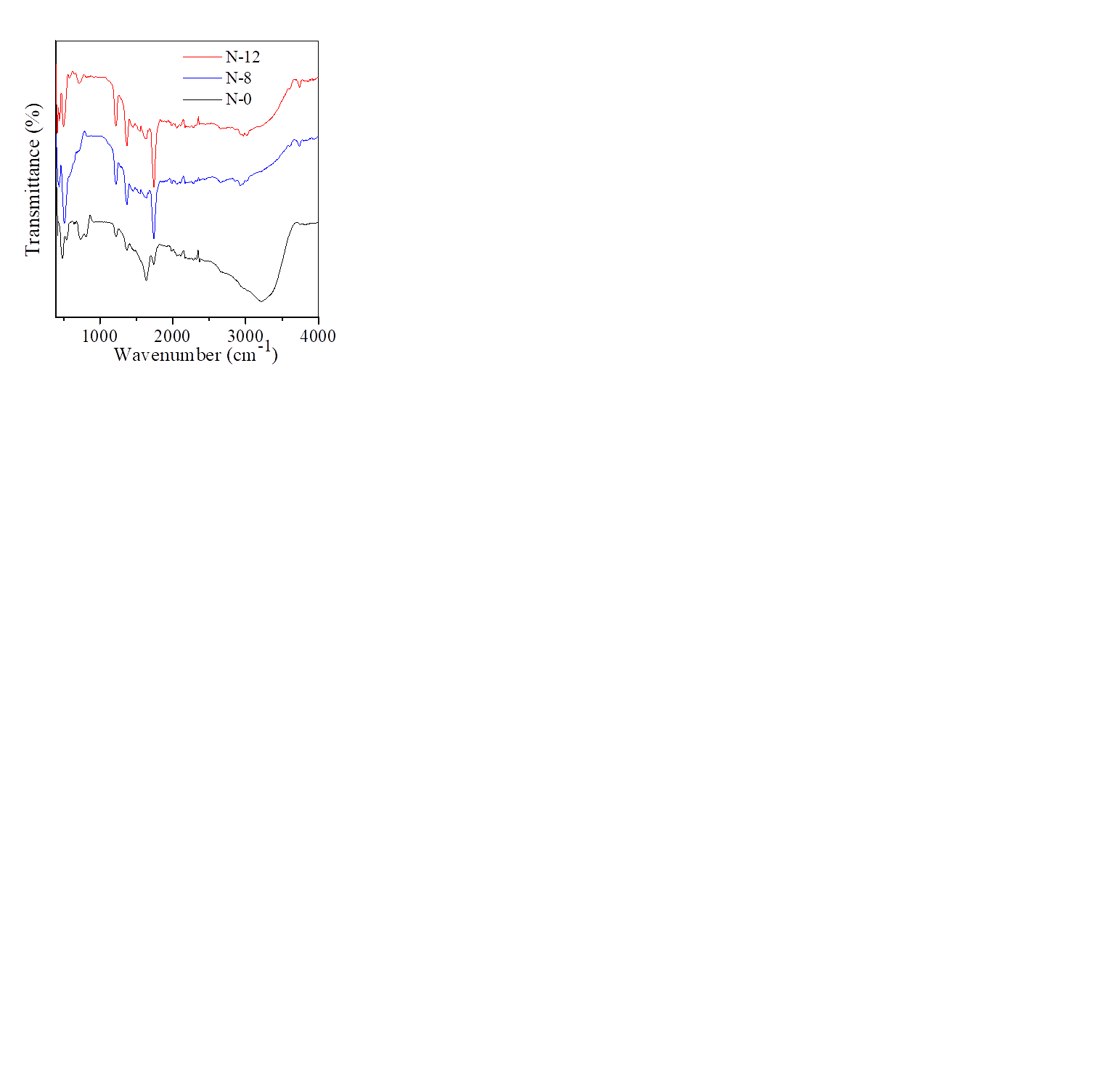


Figure S4. FTIR spectra of as-obtained N-0, N-8 and N-12 nanomaterials.

Table S3. Deconvoluted N 1s peak areas and their respective percentage of N-1, N-8 and N-12.

| Catalysts | ^a^Peak Area of N_sub_ | ^a^Peak Area of N_int_ | ^a^Peak Area of [[N_ads_](mailto:Nmol@401.5eV)](mailto:Nmol@401.5eV) | ^b^Amount N_sub_ (%) | ^b^Amount N_int_ (%) | ^b^Amount [N_ads_](mailto:Nmol@401.5eV) (%) |
| --- | --- | --- | --- | --- | --- | --- |
| N-1 | 14.71 | 161.22 | 23.42 | 7.38 | 80.87 | 11.75 |
| N-8 | 8.17 | 223.00 | 6.52 | 3.44 | 93.82 | 2.74 |
| N-12 | 9.8 | 336.21 | 24.86 | 2.65 | 90.65 | 6.70 |

*^a^peak area is the individual fitted peak area;*

*^b^amount is the individual percentage of fitted peak area;*

*N_sub_ is substitutional nitrogen;*

*N_int_ is interstitial nitrogen;*

*N_ads_ is chemisorbed nitrogen*

Table S4. The reaction rate constant (k) of MB degradation in the presence of various pure and N/TiO_2_ samples under visible-light illumination

| Catalysts | k (10^-2^ min^-1^) | R^2^ |
| --- | --- | --- |
| MB | 0.101 | 0.95 |
| N-0 | 0.194 | 0.98 |
| N-1 | 1.165 | 0.99 |
| N-4 | 1.266 | 0.99 |
| N-8 | 1.257 | 0.99 |
| N-12 | 3.252 | 0.99 |

Table S5. Comparison of synthesis and photocatalytic performance of various N/TiO_2_ materials.

| N/TiO_2_ photocatalyst | Visible Light Source | Irrad. Time | Performance | Remark | Ref. |
| --- | --- | --- | --- | --- | --- |
| Method: Sol-gel  N dopant: guanidine chloride  N/Ti: 1:1  Phase: anatase, rutile  E_g_ or λ_absorbed_: 2.91eV  S_BET_: 80 m^2^/g | 112 W Fluorescent lamps | 1.67 hr | 97% with 0.033/min | Catalyst: 50 mg  MB Conc.: 10 mg/L  Total Vol.: 100 mL | Current Study |
| Method: Hydrothermal  N dopant: ethylenediamine  N/Ti: 1:1  Phase: anatase  E_g_ or λ_absorbed_: ~460 nm  S_BET_: 172 m^2^/g | Xenon lamp | 1 hr | 92% | Catalyst: 100 mg  MB Conc.: 10 mg/L  Total Vol.: 100 mL | ^[5]^ |
| Method: Sol-gel  N dopant: triethylamine  N/Ti: 3%  Phase: anatase, rutile  E_g_ or λ_absorbed_: ~2.98 eV  S_BET_: 88 m^2^/g | 150 W Xenon lamp | 7 hr | 0.007/min | Catalyst: 20 mg  MB Conc.: 10^-2^ M  Total Vol.: 50 mL | ^[6]^ |
| Method: Sol-gel  N dopant: ethylene-  diaminetetraacetic acid  N/Ti: 3:1  Phase: anatase, rutile  E_g_ or λ_absorbed_: 2.98 eV  S_BET_: 80 m^2^/g | Solar simulator with blue filter | 3 hr | 0.038/min | Catalyst: 60 mg  MB Conc.: 10 ^-5^ M  Total Vol.: 50 mL | ^[7]^ |
| Method: Chemical method  N dopant: ammonia  N/Ti: not reported  Phase: anatase  E_g_ or λ_absorbed_: ~500 nm  S_BET_: 73 m^2^/g | 400 W Mercury lamp | 0.5 hr | N/TiO_2_ shows higher activity than the Degussa P25 | Catalyst: 25 mg  MB Conc.: 110 ppm  Total Vol.: 25 mL | ^[8]^ |
| Method: Hydrothermal  N dopant: DMF  N/Ti: 2:1  Phase: anatase, rutile,brookite  E_g_ or λ_absorbed_: 2.63 eV  S_BET_: 136 m^2^/g | 500 W Xenon lamp | 2 hr | 90% | Catalyst: 62.5 mg  MB Conc.: 10 mg/L  Total Vol.: 50 mL | ^[9]^ |
| Method: Sol-gel- microwave  N dopant: urea  N/Ti: 20%  Phase: anatase, rutile  E_g_ or λ_absorbed_: 1.95 eV  S_BET_: not reported | 30 W Fluorescent lamp | 5 h | 73% with 0.3/hr | Catalyst: 150 mg  MB Conc.: 10 mg/L  Total Vol.: 50 mL | ^[10]^ |
| Method: Hydrothermal  N dopant: guanidine carbonate  N/Ti: 1:2  Phase: anatase, monoclinic  E_g_ or λ_absorbed_: 500 nm  S_BET_: 6 m^2^/g | 10 W Fluorescent lamps | 1 hr | 80% | Catalyst: 37.5 mg  MB Conc.: 1x10^-5^ M | ^[11]^ |

Figure S5. Recycle test of N-12 photocatalyst under 100 min visible-light illumination.


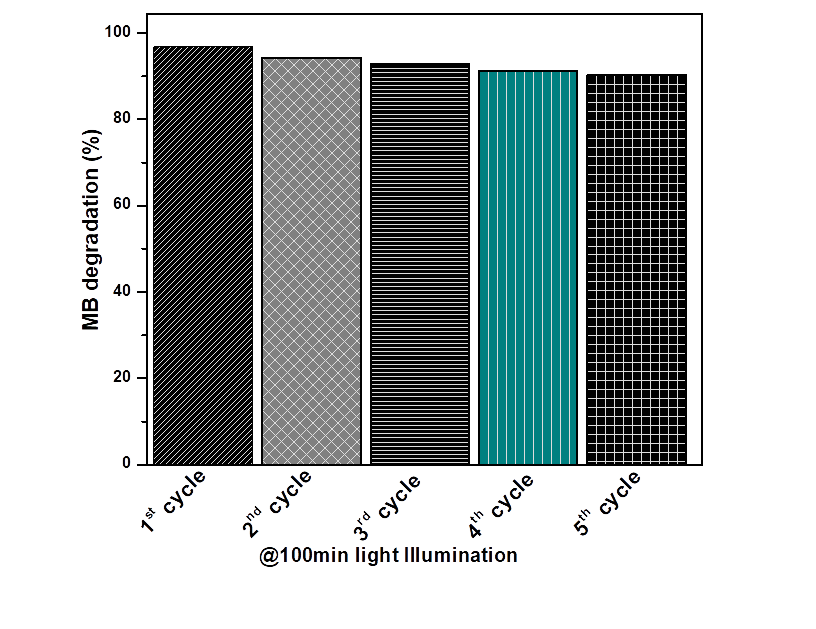


# S2. The thermogravimetric analysis of as-prepared TiO_2_ based catalysts

The thermogram (Figure S6) of bare and N/TiO_2_ samples reveal that the mass difference around 100-200 ^o^C and 300-450 ^o^C is attributed to the loss of physically adsorbed water and nitrogen and/or carbon related species, respectively. It can be noted that the N-12 exhibited a better thermal stability with weight retention of 94% than unmodified titania and less-days aged N/TiO_2_ samples.


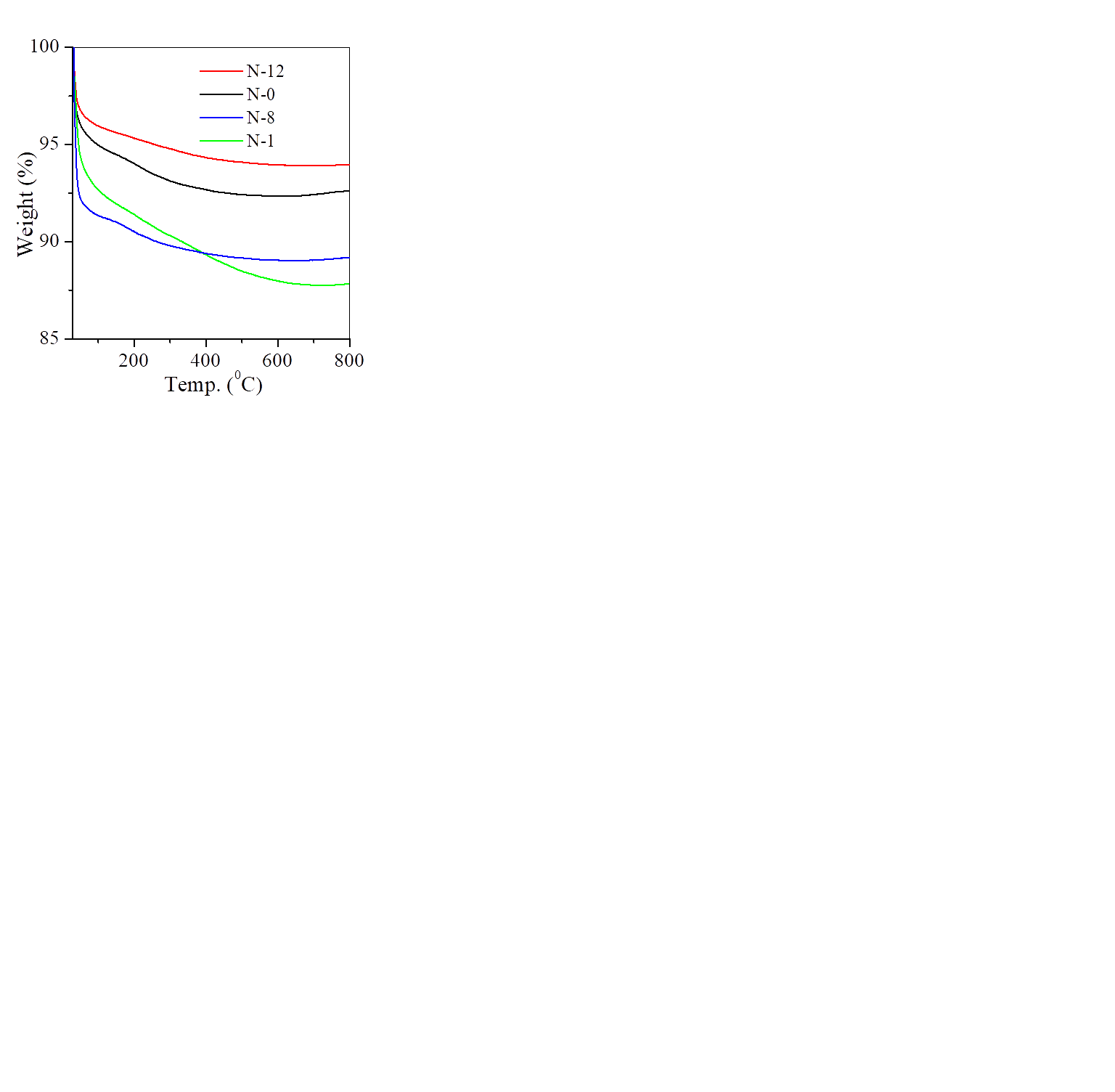


Figure S6. Thermogram of as-obtained nanomaterials.


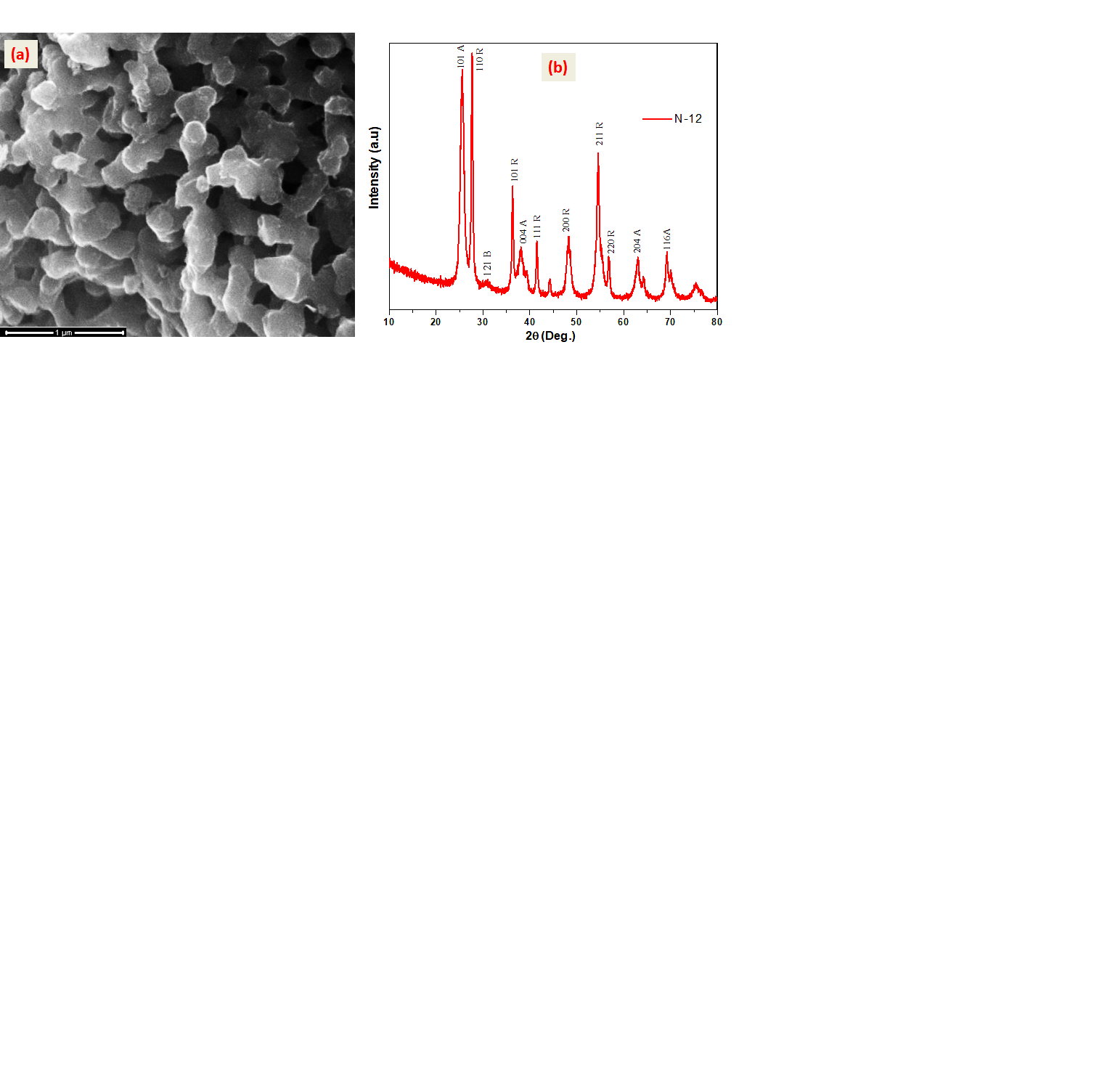


Figure S7. FESEM image (a) and XRD pattern (b) of N-12 photocatalyst after five MB degradation tests; A=Anatase, R=Rutile, B=Brookite.


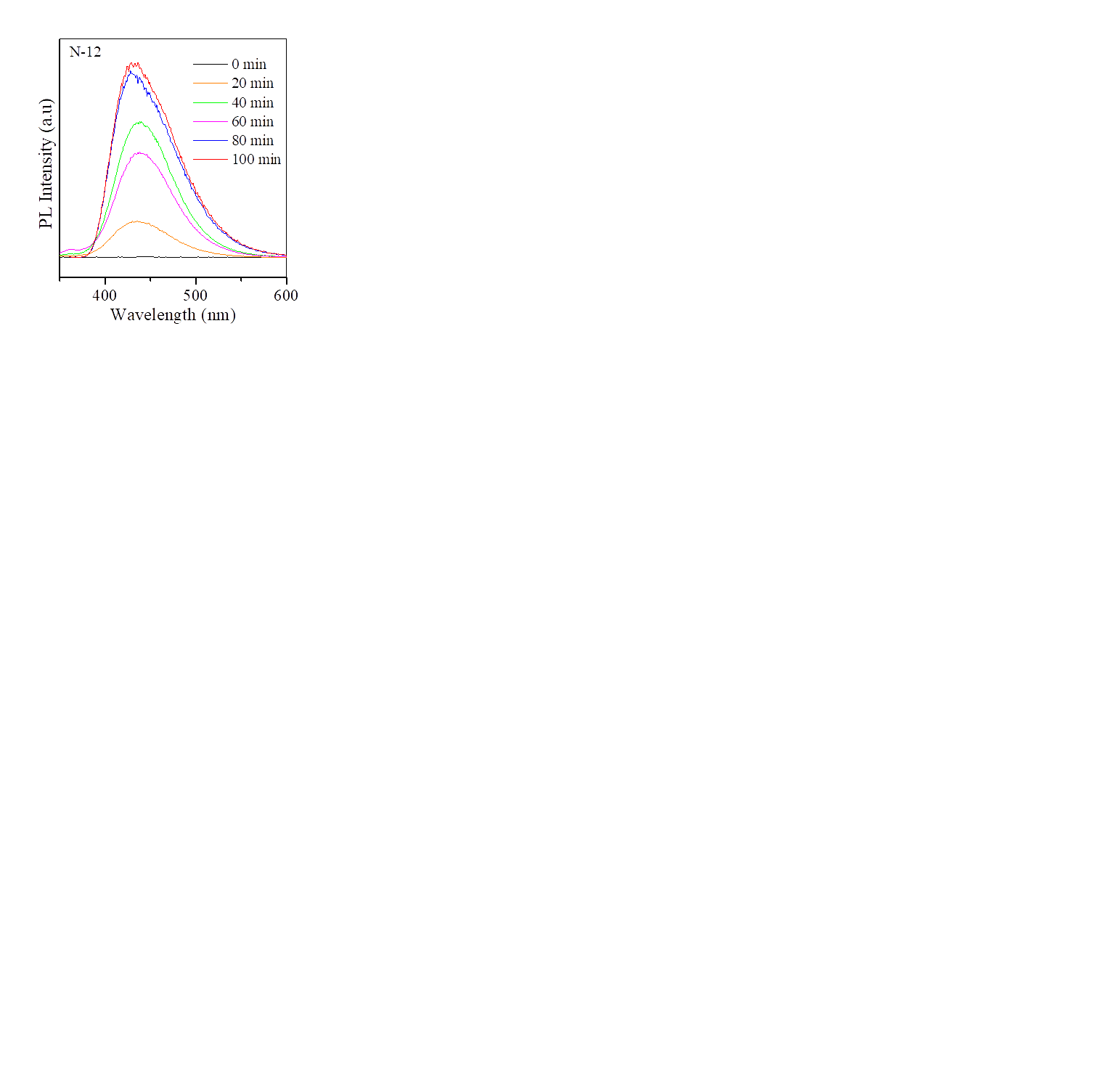


Figure S8. Fluorescence intensity of 2-hydroxyterephthalic acid solution for N-12 sample under different visible-light irradiation

Figure S9. Photodegradation mechanism of MB over surface of photocatalyst.^[12–14]^

# S3. Calculating CB/VB positions of as-prepared N/TiO_2_ catalyst

The CB and VB positions of N/TiO_2_ were calculated using the following equations:^[15]^

$$E_{VB}=X-E_{e}+0.5E_{g} (1)$$

$$E_{CV}=E_{VB}-E_{g} (2)$$

where E_VB_/E_CB_ are the VB and CB potentials, respectively relative to NHE, X is the electronegativity of the semiconductor, for TiO_2_ is 5.81, E_e_ is the energy of free electrons (4.5 eV) and E_g_ is the band gap energy, 2.92 eV for N-12. Accordingly, it is found that the E_CB_ and E_VB_ of N-12 are -0.145 and 2.77 eV, respectively.

# S4. Characterization Techniques

The structure and phase purity of the as-obtained materials were obtained by Powder X-ray Diffraction (XRD) using a X-ray diffractometer (Bruker D8 Advance, Cu Kα 0.15406 nm, 40 kV, 30 mA) at a scan rate of 2^o^ min^-1^. Material Diffraction Analysis (MAUD) software was used for structural refinement; the reflection peaks were verified at scanning speed of 0.5^o^ min^-1^. The average crystallite size (D) of the nanomaterials was calculated using Debye-Scherrer’s equation. Raman spectra and images were analyzed by confocal Raman Microscopy (Alpha 300R, Witec Inc. Germany) with a wavelength of 633 nm laser excitation; a calibration with a silicon standard (Raman peak cantered at 520 cm^−1^) was done prior to every measurement. For the data evaluation, WITec Project plus (v 4.1) software was used. X‐ray photoelectron spectroscopy (XPS) measurements were performed using PHI 5000 Versa Probe II (ULVAC-PHI Inc., USA) equipped with micro-focused (200 μm, 15 KV monochromatic Al-Kα X-Ray source, hν = 1486.6 eV). Survey scan was carried out with X-ray power of 23.7 W at a pass energy of 187.85 eV. High-resolution spectra of the major elements were recorded at 46.95 eV pass energy; the recorded spectral analysis was done by curve fitting software XPSSPEAK 41. The electronic paramagnetic resonance (EPR) spectra were carried out by JES-FA200 spectrometer. The Fourier Transform Infrared Spectroscopy (FTIR) spectra were verified on a Perkin Elmer Spectrum Two FTIR spectrometer instrument in the scanning range of 4000-400 cm^-1^ using Attenuated Total Reflectance mode with a resolution of 4 cm^-1^ accumulating 24 scans. Morphology of as-prepared catalysts was studied by high-resolution scanning electron microscopy (FESEM, Quanta 400 FEG SEM) and high-resolution transmission electron microscopy (HRTEM, Tecnai G^2^, FEI, 300 kV, The Netherlands). Their elemental mapping was done using energy dispersive X-ray spectroscopy (EDS) that was coupled with a SEM (Carl Zeiss, Germany). UV-vis diffuse reflectance spectra (DRS) of the as-obtained photocatalysts were recorded using Jasco V-650 spectrophotometer with BaSO_4_ as reference, and automatically converted to Kübelka-Münk formalism. N_2_ adsorption-desorption isotherms were collected using Micrometrics ASAP 2020 BET analyzer; the specific surface area and pore-size distribution were calculated by Brunauer-Emmett-Teller (BET) equation and Barrett-Joyner-Halenda (BJH) method, respectively. Their thermal behaviours were analysed by a thermal analyser (STA 7300 Thermal Analysis System, Hitachi) heating to 800 ^o^C at 10 ^o^C min^-1^ heating rate. Fluorescence spectrophotometer (JASCO FP-6500) was used at excitation wavelength of 330 nm to obtain the photoluminescence (PL) spectra of photocatalysts.

# References

[1] A. T. Kuvarega, R. W. M. Krause, B. B. Mamba, *J. Phys. Chem. C* **2011**, *115*, 22110–22120.

[2] M. Xing, X. Li, J. Zhang, *Sci. Rep.* **2014**, *4*, 1–7.

[3] W. Balcerski, S. Y. Ryu, M. R. Hoffmann, *J. Phys. Chem. C* **2007**, *111*, 15357–15362.

[4] Y. Hirose, T. Mori, Y. Morishita, A. Itadani, T. Kudoh, T. Ohkubo, T. Matsuda, S. Kittaka, Y. Kuroda, *Inorg. Chem.* **2011**, *50*, 9948–9957.

[5] G. Yang, Z. Jiang, H. Shi, Z. Yan, *J. Mater. Chem.* **2010**, *20*, 5301–5309.

[6] R. Jaiswal, J. Bharambe, N. Patel, A. Dashora, D. C. Kothari, A. Miotello, *Appl. Catal. B Environ.* **2015**, *168*–*169*, 333–341.

[7] V. Etacheri, M. K. Seery, S. J. Hinder, S. C. Pillai, *Chem. Mater.* **2010**, *22*, 3843–3853.

[8] M. Sathish, B. Viswanathan, R. P. Viswanath, C. S. Gopinath, *Chem. Mater.* **2005**, *17*, 6349–6353.

[9] H. Wang, X. Gao, G. Duan, X. Yang, X. Liu, *J. Environ. Chem. Eng.* **2015**, 1–6.

[10] J. Zhang, L. J. Xu, Z. Q. Zhu, Q. J. Liu, *Mater. Res. Bull.* **2015**, *70*, 358–364.

[11] S. Chainarong, L. Sikong, S. Pavasupree, *Energy Procedia* **2011**, *9*, 418–427.

[12] Q. Wang, S. Tian, P. Ning, *Ind. Eng. Chem. Res.* **2014**, *53*, 643−649.

[13] C. Yang, W. Dong, G. Cui, Y. Zhao, X. Shi, X. Xia, B. Tang, W. Wang, *RSC Adv.* **2017**, *7*, 23699–23708.

[14] A. Houas, H. Lachheb, M. Ksibi, E. Elaloui, C. Guillard, J. M. Herrmann, *Appl. Catal. B Environ.* **2001**, *31*, 145–157.

[15] G. Gebreslassie, P. Bharali, U. Chandra, A. Sergawie, P. K. Boruah, M. R. Das, E. Alemayehu, *J. Photochem. Photobiol. A Chem.* **2019**, *382*, 111960.
